# The role of the size in thyroid cancer risk stratification

**DOI:** 10.1038/s41598-021-86611-6

**Published:** 2021-03-31

**Authors:** Federica Vianello, Simona Censi, Sara Watutantrige-Fernando, Susi Barollo, Yi Hang Zhu, Nora Albiger, Loris Bertazza, Jacopo Manso, Sofia Carducci, Clara Benna, Maurizio Iacobone, Francesca Galuppini, Gianmaria Pennelli, Caterina Mian

**Affiliations:** 1 Department of Radiotherapy, Istituto Oncologico Veneto-IRCCS, Padua, Italy; 2 Endocrinology Unit, Department of Medicine (DIMED), University of Padua, Padua, Italy; 3 Family Cancer Clinic, Istituto Oncologico Veneto-IRCCS, Padua, Italy; 4 Surgery Unit, Department of Surgical, Oncological and Gastroenterological Sciences (DiSCOG), University of Padua, Padua, Italy; 5 Endocrine Surgery Unit, Department of Surgical, Oncological and Gastroenterological Sciences (DiSCOG), University of Padua, Padua, Italy; 6 Surgical Pathology and Cytopathology Unit, Department of Medicine (DIMED), University of Padua, Padua, Italy

**Keywords:** Thyroid diseases, Cancer genetics

## Abstract

Only a minority of cases of differentiated thyroid carcinoma (DTC) have a poor clinical outcome. Clinical outcomes and molecular aspects were assessed in: 144 DTC ≤ 40 mm without distant metastases (group 1); 50 DTC > 40 mm without distant metastases (group 2); and 46 DTC with distant metastases (group 3). Group 3 had a worse outcome than the other two groups: during the follow-up, patients more frequently had persistent disease, died, or underwent further treatment. The outcomes did not differ between groups 1 and 2. Group 3 had a higher prevalence of *TERT* promoter mutations than group 2 (32.6% vs 14%). Group 1 had a higher frequency of *BRAF* mutations than groups 2 or 3 (61.1% vs 16.0% and 26.1%, respectively), while *RAS* mutations were more common in group 2 than in groups 1 and 3 (16.0% vs 2.1% and 6.5%, respectively). Groups 1 and 2 shared the same outcome, but were genetically distinct. Only lymph node involvement, distant metastases, older age and (among the molecular markers) *TERT* promoter mutations were independent predictors of a worse outcome. Metastatic DTC had the worst outcome, while the outcome was identical for large and small non-metastatic DTC, although they showed different molecular patterns. *TERT* promoter mutations emerged as an independent factor pointing to a poor prognosis.

## Introduction

Differentiated thyroid cancer (DTC), which includes papillary and follicular thyroid cancer (PTC and FTC, respectively), is the most common endocrine malignancy, accounting for about 3.1% of cancers in 2018. Its worldwide incidence has been increasing rapidly in the last four decades, and the age-standardized mortality rate for DTC is estimated at about 0.42 per 100,000 population a year^1,2^.

DTC is characterized by an excellent prognosis, with a 5-year overall survival rate of about 97% for PTC, and 89% for FTC. Some patients nonetheless develop aggressive tumors with poor clinical outcomes. The most important clinical and pathological features conferring a more aggressive phenotype are: age at diagnosis; primary tumor size; soft tissue invasion; and distant metastases (found in about 2–5% of cases)^3–5^. These variables, and other risk factors are weighted differently in the numerous staging systems available (such as the European Organization for Research and Treatment of Cancer [EORTC]; the Age, Grade, Extent, Size [AGES]; the Age, Metastases, Extent, Sex [AMES]; the Metastases, Age, Completeness of resection, Invasion, Size [MACIS] criteria; the Memorial Sloan Kettering Cancer Center [MSKCC] or the National Thyroid Cancer Treatment Cooperative Study [NTCTCS] systems), depending on the prognostic importance attributed to them, but none of these approaches have proved clearly superior. The 2009 version of the American Thyroid Association’s guidelines strongly support risk classification, considering not only the mortality risk, but also the risk of recurrent and persistent structural disease. The latter risk has a greater impact in thyroid cancer management, given that disease-specific mortality is usually low. The American Joint Committee on Cancer (AJCC) staging system was developed primarily to predict mortality risk, however, and (even now in its 8th edition) tumor extension and distant metastases have remained as prognostic factors over the years, while the age threshold of prognostic significance has shifted up to 55 years^6–8^. In the TNM classification, a larger tumor size, in the absence of signs of its extension into other tissues, is not considered a negative prognostic factor for survival. Some risk classifications (like the MSKCC, AGES and MACIS) suggest otherwise, and some large studies have shown that size can affect patient survival^4,9–11^, and may be a predictor of distant metastasis^12^. In their recent monocentric series of 5897 patients with a median follow-up of 177 months, Ito et al*.* found cancer > 4 cm in size an independent predictor of a worse cause-specific survival (CSS), together with metastatic disease and older age^4^. In short, there is still uncertainty about the influence of tumor size on patient outcome and survival, and large tumors have not been sufficiently investigated in the literature.

In the last few years, much effort has gone into characterizing the molecular drivers responsible for the onset of DTC in an attempt to clarify patients’ prognosis. The mutations most often considered concern *BRAF* and *RAS*, and—more recently—the telomerase reverse transcriptase (*TERT)* promoter. A great body of evidence has demonstrated that *TERT* promoter mutations in DTC are associated with a higher stage at diagnosis, and with distant metastases (even when they are only discovered at cytology)^13–15^. More importantly, *TERT* promoter mutations are an independent factor for predicting patient mortality^16^ and disease-free survival (DFS)^13,15,17^.

A greater knowledge of the clinical, pathological and molecular features of DTC might improve the diagnostic frame and lead to customized therapies. On these premises, the aims of the present single-center, retrospective study were: (1) the clinical, pathological and molecular characterization (based on *BRAF*, *RAS*, *TP53*, *PTEN*, *PIK3CA* genes, and *TERT* promoter analysis) of selected cases of DTC with or without distant metastases, grouped by primary tumor size: group 1 included patients with tumors ≤ 40 mm in size and no distant metastases; group 2 included patients with tumors > 40 mm in size, with no distant metastases; and group 3 included patients with metastatic DTC, regardless of tumor size.

## Results

### Clinical and pathological characteristics, and outcomes

The clinical characteristics of the three groups are summarized in Table 1. The sample of patients included: 144/240 (60%) in group 1, 50/240 (20.8%) in group 2, and 46/240 (19.2%) in group 3.

**Table 1:** Clinical-pathological characteristics of the three groups.

	Group 1 (n. 144)	Group 2 (n. 50)	Group 3 (n. 46)	*p*-value
Sex, F:M	118:26 (4.5:1)	30:20 (1.5:1)	22:24 (0.9:1)	< 0.01
**Age at diagnosis (years)**				< 0.01
Median	47.0	45.5	61.9	
Range	21.6–80.8	22.0–92.0	23.9–78.0	
**Size (mm)**				< 0.01
Median	13	49	26.5	
Range	2 – 38	42–85	2–90	
**T stage**				< 0.01
X	0/144 (0.0%)	0/50 (0.0%)	4/46 (8.7%)	
1	122/144 (84.7%)	0/50 (0.0%)	15/46 (32.6%)	
2	22/144 (15.3%)	0/50 (0.0%)	8/46 (17.4%)	
3	0/144 (0.0%)	48/50 (96.0%)	11/46 (23.9%)	
4	0/144 (0.0%)	2/50 (4.0%)	8/46 (17.4%)	
**N stage**				< 0.01
X	14/144 (9.7%)	29/50 (58.0%)	12/46 (26.1%)	
0	78/144 (54.2%)	13/50 (26.0%)	10/46 (21.7%)	
1	52/144 (36.1%)	8/50 (16.0%)	24/46 (52.2%)	
**M stage**				< 0.01
0	144/144 (100%)	50/50 (100%)	0/46 (0%)	
1	0/144 (0.0%)	0/50 (0.0%)	46/46 (100%)	
Metastases at time of diagnosis			30/46 (65.2%)	
Metastases developing during follow-up			16/46 (34.8%)	
**Disease stage**				< 0.01
I	131/144 (91.0%)	30/50 (60.0%)	0/46 (0.0%)	
II	13/144 (9.0%)	20/50 (40.0%)	15/46 (32.6%)	
III	0/144 (0.0%)	0/50 (0.0%)	0/46 (0.0%)	
IV	0/144 (0.0%)	21/50 (0.0%)	31/46 (67.4%)	
**Histotype**				< 0.01
PTC	144/144 (100%)	34/50 (68.0%)	35/46 (70.1%)	
FTC	0/144 (0.0%)	16/50 (32.0%)	11/46 (29.9%)	
WI-FTC		6/16 (37.5%)	8/11 (72.7%)	
MI-FTC		10/16 (62.5%)	3/11 (27.3%)	
**Multifocality**	80/144 (55.6%)	15/50 (34.0%)	21/36 (58.3%)	0.02
**Follow-up (months)**				< 0.01
Median	81.8	51.7	94.9	
Range	17.7–130.6	12.9–134.9	32.5–237	
Second treatment	8/144 (5.6%)	4/50 (9.0%)	36/45 (80.0%)	< 0.01
**Disease status**				< 0.01
Remission	131/144 (90.9%)	44/50 (88.0%)	10/46 (21.7%)	
Biochemical incomplete response	5/144 (3.5%)	2/50 (4.0%)	4/46 (8.8%)	
Indeterminate response	6/144 (4.2%)	3/50 (6.0%)	0/46 (0.0%)	
Structural incomplete response	2/144 (1.4%)	1/50 (2.0%)	22/46 (47.8%)	
Disease-related death	0/144 (0.0%)	0/50 (0.0%)	10/46 (21.7%)	

*PTC* papillary thyroid carcinoma, *FTC* follicular thyroid carcinoma, *WI-FTC* widely-invasive follicular thyroid carcinoma, *MI-FTC* minimally-invasive follicular thyroid carcinoma, *FU* follow-up.

**Figure 1:**
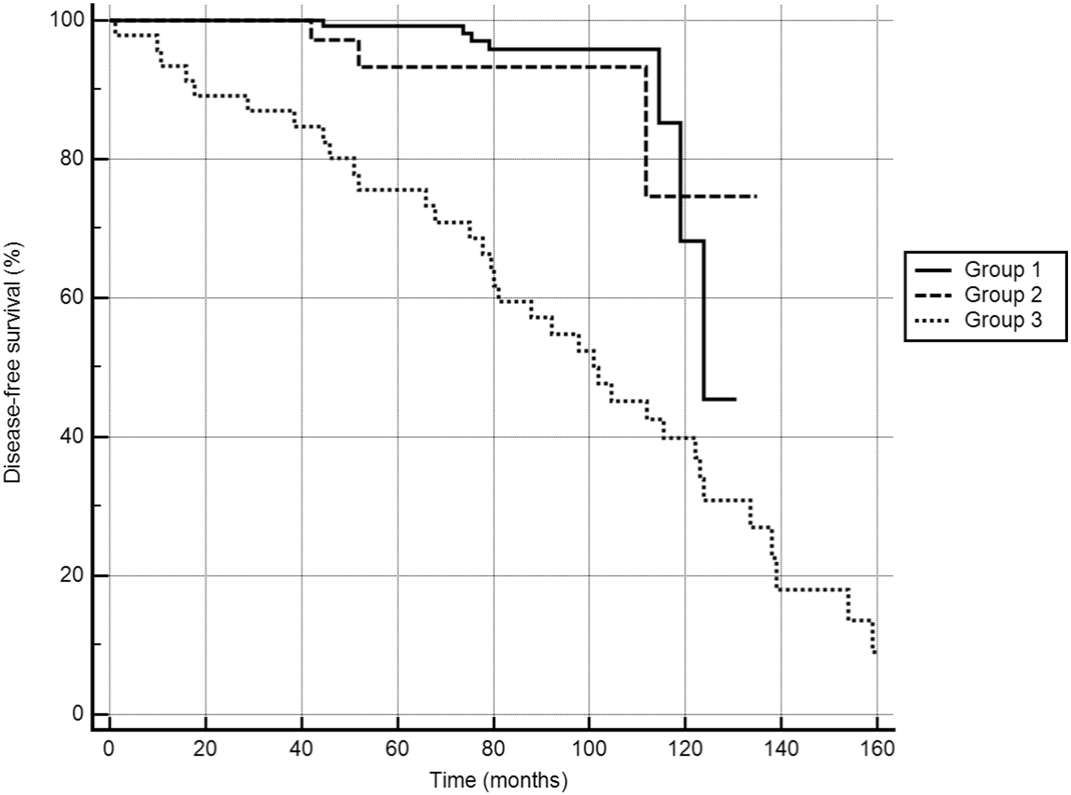
Disease-free survival in the three groups (p < 0.01), MedCalc Statistical Software version 19.1.7 (MedCalc Software Ltd, Ostend, Belgium; https://www.medcalc.org; 2020).

**Figure 2:**
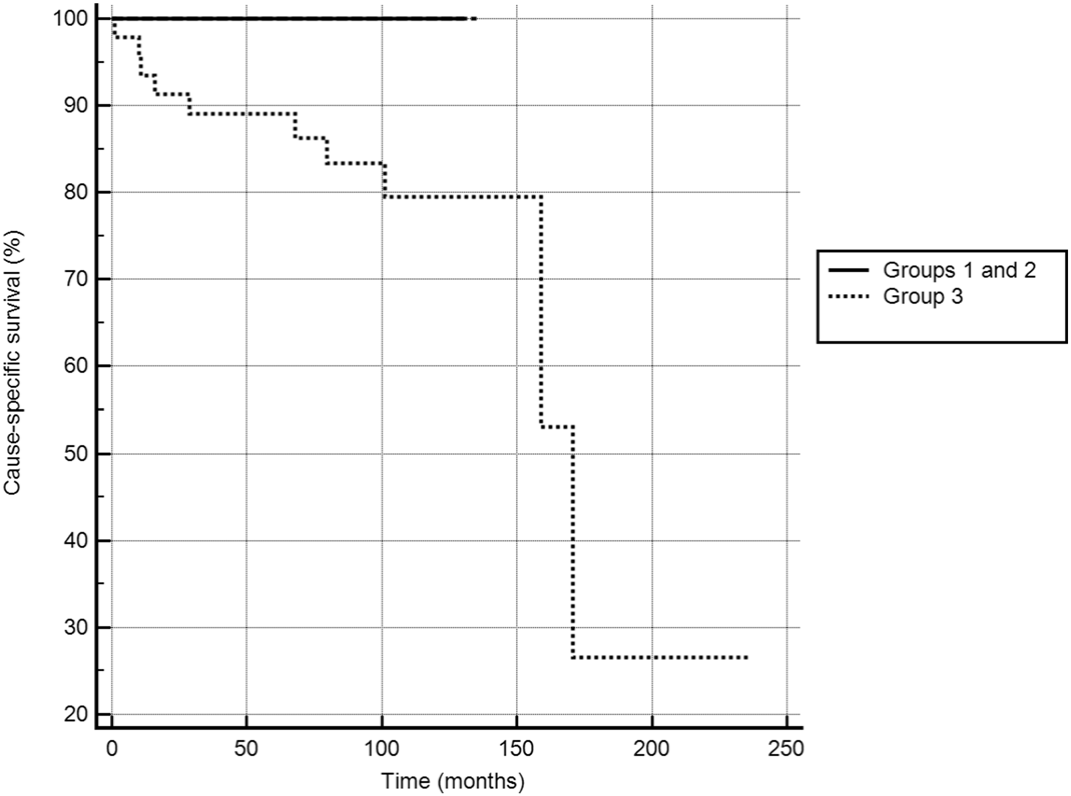
Cause-specific survival in the three groups (p < 0.01), MedCalc Statistical Software version 19.1.7 (MedCalc Software Ltd, Ostend, Belgium; https://www.medcalc.org; 2020).

The patients in group 3 were more frequently males (p < 0.01), and were older than those in the other two groups (p < 0.01). Group 3 included a higher proportion of cases of widely invasive FTC (WI-FTC), with 72.7% in group 3 and 37.5% in group 2 (there were no cases of FTC in group 1). Patients in group 3 were also more likely to undergo a second treatment (80.0% versus 9.0% in group 2, and 5.6% in group 1, p < 0.01), and to have a worse outcome, with higher rates of persistence/recurrence or death (78.2% versus 6.0% in group 2, and 4.9% in group 1, *p* < 0.01). Patients with metastatic tumors (group 3) had a shorter DFS (p < 0.01) (Fig. 1), and a worse CSS (p < 0.01) (Fig. 2) then the other two groups. No differences in patient outcomes emerged between groups 1 and 2.

#### Molecular characteristics

The molecular characteristics of the three groups are summarized in Table 2.

**Table 2:** Molecular characteristics of the three groups (also by histology).

	Mutation	Group 1 (n. 144)	Group 2 (n. 50)	Group 3 (n. 46)	*p*-value
	*BRAF*	88/144 (61.1%)	8/50 (16.0%)	12/46 (26.1%)	< 0.01
	*RAS*	3/144 (2.1%)	8/50 (16.0%)	3/46 (6.5%)	0.006
	*TERT*	5/133 (3.8%)	7/50 (14.0%)	15/46 (32.6%)	< 0.01
	*TERT*	2/5 (40.0%)	2/7 (28.6%)	11/15 (73.3%)	< 0.01
	*TERT + BRAF*	3/5 (60.0%)	1/7 (14.3%)	2/15 (13.3%)	
	*TERT + RAS*	0/5 (0.0%)	3/7 (42.8%)	1/15 (6.7%)	
	*TERT + PTEN*	0/5 (0.0%)	1/7 (14.3%)	0/15 (0.0%)	
	*TERT + BRAF + PIK3A*	0/5 (0.0%)	0/7 (0.0%)	1/15 (6.7%)	
	*TP53*	1/133 (0.8%)	1/44 (2.3%)	0/44 (0.0%)	0.11
	*PTEN*	0/144 (0.0%)	1/50 (2.0%)	0/45 (0.0%)	0.09
	*PIK3CA*	0/144 (0.0%)	1/43 (2.3%)	2/40 (5.0%)	0.02

PTC (n. 213)		n. 144	n. 34	n. 35	
	*BRAF*	88/144 (61.1%)	7/34 (20.6%)	10/35 (28.6%)	< 0.01
	*V600E*	87/88 (98.9%)	7/7 (100%)	10/10 (100%)	
	*K601E*	1/88 (1.1%)	0/7 (0.0%)	0/10 (0.0%)	
	*RAS*	3/144 (2.1%)	7/34 (20.6%)	1/35 (2.9%)	< 0.01
	*TERT*	5/133 (3.8%)	2/34(5.9%)	10/35 (28.6%)	< 0.01
	*TERT*	2/5 (40.0%)	0/2 (0.0%)	9/10 (90.0%)	
	*TERT + BRAF V600E*	3/5 (60.0%)	1/2 (50.0%)	1/9 (10.0%)	
	*TERT + RAS*	0/5 (0.0%)	1/2 (50.0%)	0/9 (0.0%)	
	*TP53*	1/133 (0.8%)	1/30 (3.3%)	0/33 (0.0%)	
	*PTEN*	0/144 (0.0%)	0/33 (0.0%)	0/35 (0.0%)	
	*PIK3CA*	0/144 (0.0%)	1/29 (3.4%)	0/28 (0.0%)	

FTC (n. 27)		n. 0	n. 16	n. 11	
	*BRAF K601E*	–	1/16 (6.2%)	2/11 (18.2%)	
	*RAS*	–	1/16 (6.2%)	2/11 (18.2%)	
	*TERT*	–	5/16 (31.2%)	5/11 (45.5%)	
	*TERT*		2/5 (40.0%)	2/5 (40.0%)	
	*TERT + BRAF K601E*		1/5 (20.0%)	1/5 (20.0%)	
	*TERT + RAS*		1/5 (20.0%)	1/5 (20.0%)	
	*TERT + PTEN*		1/5 (20.0%)	0/5 (0.0%)	
	*TERT + BRAF K601E + PIK3CA*		0/5 (0.0%)	1/5 (20.0%)	
	*TP53*		0/14 (0.0%)	0/11 (0.0%)	
	*PTEN*		1/16 (6.2%)	0/11 (0.0%)	
	*PIK3CA*		0/14 (0.0%)	2/11 (18.2%)	

### *BRAF* mutations

*BRAF* mutations were detected in 88/144 (61.1%) of patients in group 1, 8/50 (16.0%) in group 2, and 12/46 (26.1%) in group 3 (p < 0.01). The *BRAF* mutation was significantly more frequent in group 1 than in the other two groups (p < 0.01). The type of *BRAF* mutation was related to the histotype: *BRAF V600E* was detected in all but one PTC, while *BRAF K601E* was the only *BRAF* mutation found in cases of FTC.

### *RAS* mutations

*RAS* mutations were detected in 3/144 (2.1%) cases in group 1, 8/50 (16.0%) in group 2, and 3/46 (6.5%) in group 3. They were more common in large, non-metastatic tumors than in the other two groups (p = 0.006).

### *TERT* promoter mutations

*TERT* promoter mutations were detected in 5/133 (3.8%) patients in group 1, 7/50 (14.0%) in group 2, and 15/46 (32.6%) in group 3, and the rising frequency from group 1 to group 3 was statistically significant (p < 0.01). The *TERT* promoter showed the *C228T* mutation in 23/27 cases (85.2%), and the C250T mutation in 4/27 (14.8%).

As concerns the histotype, 17/27 (62.9%) patients with a *TERT* promoter mutation had PTC, while the other 10/27 (37.1%) had FTC.

Among the 27 patients with *TERT* mutations, 8 (29.6%) also had *BRAF* mutations, and 4/27 (14.8%) also had *RAS* mutations. In one case (of WI-FTC), we found a homozygous *TERT* promoter mutation together with *BRAFK601E* and *PIK3CA* mutations (this case had been reported in 2011^18^, but *TERT* analysis had not been done at the time). A combination of *TERT* and *PTEN* mutations was also found in one case of large FTC without distant metastases.

### *TP53, PTEN* and *PIK3CA* mutations

A *TP53* mutation was found on exon 6 in only 1/133 patients (0.8%) in group 1 (*G293R*), and in 1/44 (2.3%) in group 2 (*D208N*). *PTEN* mutations were detected on exon 5 in 1/50 patients (2%) in group 2 (*D93N*). *PIK3CA* mutations were detected in 1/43 patients (2.3%) in group 2 (*R516K*) who had PTC, and in 2/40 (5.0%) of patients in group 3 (one carrying an *E545A* mutation, the other carrying a *H1047R* mutation), both cases of WI-FTC.

#### Clinical and molecular correlations

In the series as a whole, and within the three groups, *RAS*, *PTEN*, *TP53* and *PIK3CA* mutations were not associated with patient outcomes.

*BRAF* mutations were also unassociated with outcomes in the series as a whole, but in group 1 they were weakly associated with a worse prognosis. Indeed, of the 137 patients with a remission or indeterminate response at the end of the follow-up, 81 (59.1%) were *BRAF*-mutated, as opposed to 7/7 (100%) with persistence of disease (p = 0.03).

*BRAF* was not associated with outcome in groups 2 or 3.

In the series as a whole, *TERT-*mutated patients were older at diagnosis (63 versus 47 years, *p* < 0.01), and had larger tumors (44 versus 16 mm, *p* < 0.01), and a greater tumor extension (T4: 11.5% versus 3.5%, *p* < 0.01). They more frequently had distant metastases (53.8% versus 15.3%, *p* < 0.01), and their disease was more advanced (Stage IV 40.7% versus 9.9%, *p* < 0.01). Accordingly, *TERT* promoter mutations was the only molecular event statistically associated with patient outcomes. *TERT*-mutated patients more frequently needed a second treatment (14/27 [51.8%] *TERT*-positive *versus* 34/202 [16.8%] *TERT*-negative patients mutated; p < 0.01), and had a worse outcome: a biochemical or structural incomplete response or disease-related death occurred in 28/202 patients (13.9%) without *TERT* promoter mutations as opposed to 18/27 (66.7%) *TERT-*mutated patients (p < 0.01). They also had a shorter DFS (*p* = 0.002).

Whether alone or in association with other molecular events, *TERT* promoter mutations correlated with the worst outcomes: they were detectable in 18/46 patients (39.1%) with persistent disease, and only in 9/183 patients (4.9%) with an excellent response (*p* < 0.01).

At univariate analysis, male sex, older age at diagnosis, type of cancer, TNM parameters, tumor stage, need for a second treatment, and *TERT* mutations were all associated with a worse outcome (*p* < 0.01).

At multivariate analysis, only *TERT* promoter mutations (odds ratio [OR] 8.7997; 95% confidence interval [CI] 1.9877–38.9567), age (OR: 1.0420; 95% CI 1.0023–1.0814), lymph node involvement (OR 4.2993; 95% CI 1.2530–14.7517), FTC histology (OR 1.6232; 95% CI 0.381–7.7926), and distant metastases (OR 40.3925; 95% CI 13.5846–120.1033) emerged as independent factors for a poor prognosis (p < 0.01) (Table 3).

**Table 3:** Odds ratios and 95% confidence intervals (CI) at logistic regression analysis, referred to the outcome (persistent disease or disease-related death).

Variable	Odds ratio	95% CI
Male sex	0.9373	0.2837–3.0966
*TERT* mutation	8.7997	1.9877–38.9567
Distant metastases	40.3925	15.5846–120.1033
Age	1.0420	1.0023–1.0814
Lymph node involvement	4.2993	1.2530–14.7517
FTC histology	1.6232	0.3381–7.7926

*TERT* promoter mutations also influenced outcomes for patients in groups 2 and 3. In group 2, patients with persistent disease or disease-related death were more frequently *TERT*-mutated than patients in remission or an indeterminate status: 2/3 (66.7%) versus 5/47 (10.6%), respectively (p = 0.0073). In group 3, none of the patients with a biochemical remission or indeterminate response (0/10) were *TERT*-mutated, while 41.7% of the patients with *TERT* promoter mutations were among those with a worse prognosis (15/36) (p = 0.01).

## Discussion

Although the mortality rate for DTC has remained stable, its incidence has been rising rapidly in recent decades due to the increasing use of neck ultrasound for thyroid diseases and other, unrelated conditions^7,19–23^. Many authors have focused on defining clinical, pathological and molecular characteristics of DTC useful for the purposes of identifying the minority of aggressive tumors associated with a higher likelihood of progression or death. This would enable customized treatment decisions and follow-up protocols, possibly right from the time of diagnosis. With this in mind, various classification systems have been proposed, such as the TNM classification (useful for predicting the risk of death) and the ATA grouping score (suitable for establishing the risk of persistence/recurrence)^6,8^. From a clinical standpoint, the presence of distant metastases and a tumor size greater than 40 mm have conventionally been associated with a poor prognosis, warranting radical surgery associated to radioactive iodine (RAI) therapy^6,7^. The impact of tumor size per se on patient mortality is still debated, however.

Given these premises, we examined a group of cases with distant metastases (regardless of primary tumor diameter), and a group with non-metastatic DTC larger than 40 mm in size, seeking to establish whether these forms of DTC, which are known to be more aggressive, share similar molecular features and clinical courses. We compared the characteristics of these “high-risk” groups with those of a group of cases of DTC not exhibiting these two features of aggressiveness. All patients had been diagnosed, treated and followed up according to the same standards of care at the same institution.

As expected, our findings confirm the impact of distant metastases on a patient’s clinical outcome^24^, as metastatic disease is associated with a significantly worse DFS and CSS. None of the patients without metastases died of their disease regardless of the size of their DTC. The presence of distant metastases was also the most influential independent prognostic factor in terms of poor prognosis, with an OR of around 40.0. Patients with distant metastases, were more often male, older at the time of their diagnosis, with lymph node involvement. They were more likely to need of a second treatment, and they had the worst outcome. Patients with large, but non-metastatic DTC shared the same outcome as those with small non-metastatic lesions. In other words, a large tumor size was not a risk factor in our series when considered alone. A shorter median follow-up for the group with large but not metastatic tumors may have influenced this result, although the 4.3-year median follow-up for this group should have been long enough for most relapses to become apparent^25^.

In recent years, several studies have suggested that certain molecular events—mainly *BRAF*, *RAS*, *TP53* and *TERT* promoter mutations, alone or in combination—may be useful for stratifying the behavior of DTC, and predicting which tumors are more likely to recur and/or be fatal^26–28^.

The *BRAF V600E* mutation is the most common molecular event in DTC (found in 40–60% of cases). Its presence has been historically associated with less-differentiated phenotypes carrying a poor prognosis, but its role is still debated^29–33^. The most important multicenter retrospective studies on the role of *BRAF* in DTC found it associated with key negative prognostic factors. Xing et al*.* reported, for instance, that it was not independently associated with cancer-related death, but it was independently associated with disease persistence/recurrence^34,35^. Some monocentric retrospective studies confirmed the *BRAF* mutation as an independent negative prognostic factor related to persistent disease^36^, and to aggressive characteristics such as loss of the ability to concentrate radioiodine, or acquisition of a capacity for glucose uptake^37^. On the other hand, prospective studies on patients who underwent fine needle aspiration biopsy for molecular definition and total thyroidectomy failed to confirm an role of *BRAF* mutations on patient outcomes^38,39^.

These different findings may stem from the fact that, particularly in the setting of prospective studies, the early detection of a *BRAF* mutation (particularly at cytology) prompts early surgery, so this genetic alteration may not have the time to confer the aggressive tumor characteristics found in retrospective studies. We confirmed the published data on the prevalence of *BRAF* mutations^34^, but only in our group 1 (61.1%), while it was markedly lower in groups 2 and 3 (16.0% and 26.1%, respectively). This is in line with reports on other metastatic DTC series in which this genetic event was not associated with the tumor’s potential to spread to distant sites^34,40^. In short, the mutation does not give the cancer cells a metastatic advantage. Our study has a possible bias, however, regarding the lack of FTCs among the consecutively-selected cases in group 1. This could have increased the frequency of *BRAF* mutations in group 1 by comparison with the other groups, since this mutation is uncommon in FTCs^41^.

Our data revealed a higher prevalence of the *RAS* mutation than reported elsewhere in the literature^34,42,43^, particularly in our group 2 (16.0% of cases revealed *RAS* mutations). We found no association between these mutations and the patients’ clinical features, probably because of the small size of this sample.

*TERT* promoter mutations in DTC were first described in 2013^44,45^. The literature has since consistently demonstrated an association between *TERT* promoter mutations and poor outcomes. This mutation in DTC patients is associated with older age, larger tumors, distant metastases, and advanced stage at diagnosis^13,16,17^. In our series too, the frequency of *TERT* promoter mutations rose with DTC aggressiveness and risk of progression: it was 3.8% group 1, 14.0% in group 2, and 32.6% in group 3.

In line with previous reports, *TERT* promoter mutations in our series as a whole were associated with older age at diagnosis, more advanced tumor stage, more frequent need for a second treatment, and worse outcomes (around 67% of patient had persistent disease or died of their disease). At multivariate analysis, *TERT* promoter mutations was the only variable independently associated with a negative prognosis.

There are reports in the literature on associations between *TERT* mutations and other oncogenic molecular events. This condition was found related to an aggressive DTC behavior, including distant metastases and a lack of radioiodine uptake capability^13,37,45^. We found no such negative impact of simultaneous mutations on the prognosis for patients with DTC, by comparison with those harboring a single *TERT* promoter mutation. It could be useful to consider larger series in order to clarify the real impact of multiple mutations. The other molecular events considered here—*PIK3CA*, *PTEN* and *TP53* mutations—seem to be rare, and almost exclusive to high-risk DTC, as previously reported^17^. It is worth noting that the presence of a *TERT* promoter mutation was able to influence the prognosis even in our group 3, with metastatic disease.

It has been reported that age, tumor size, extrathyroidal extension, and nodal and distant metastases are predictors of a patient’s mortality risk^46–51^. Our data, focusing on tumor size and distant metastases, only confirm the latter as a predictor of mortality.

It is noteworthy that patients with larger DTCs shared much the same outcome as those with smaller lesions, though they differed considerably from a molecular standpoint. *RAS* and *TERT* mutations were more common in group 2, while *BRAF* mutations were more prevalent in group 1. Group 2 had a molecular profile more similar to that of group 3, except for a higher frequency of *RAS* mutations in the former. *TERT* promoter mutations were more frequent than in group 1, but still lower than in metastatic patients. Based on our results and the conflicting data in the literature regarding its prognosis, we wonder whether large DTCs may be a sort of intermediate entity, in between small indolent DTCs and those with a poor outcome. Our data suggest a shift in the molecular profile from DTC < 4 cm in size to metastatic tumors, with large DTCs seeming to come somewhere in between, in terms of their molecular features, although they have much the same outcomes as the smaller, non-metastatic tumors. It may be that many other molecular events need to accumulate in large tumors to have any metastatic potential and affect the prognosis. The clinical importance of cancer size is still debated. Most authors have observed an indolent behavior of microcarcinomas^52^, but some have reported distant metastases already at diagnosis or during the follow-up, and a poor prognosis even for tumors less than 10–15 mm in size^53–55^. Little is known as yet about the molecular patterns of such small but aggressive DTCs. In our series, the median size of tumors in group 3 (with metastatic disease) was smaller than in group 2, and ranged very widely, from 2 up to 90 mm, although their molecular pattern and other characteristics associated with poor outcome (lymph node involvement) were very different. These data suggest that some DTCs are inherently aggressive, already developing aggressive mutations even while they remain small in size.

A better knowledge of the molecular pattern of advanced thyroid disease could help us to identify promising therapeutic targets for advanced DTC. The treatment of choice for metastatic thyroid cancer is RAI therapy, but as DTC progresses, it can lose its capacity for iodine uptake^56^, becoming refractory to RAI. Other therapies involving tyrosine kinase inhibitors can be attempted in such cases. The loss of iodine uptake capacity in some thyroid cancers has been associated with involving *BRAF V600E*^57^ or *TERT* promoter mutations, or both^58^. Phase II trials with the *BRAF V600E* inhibitors vemurafenib^59^ and dabrafenib^60^ (two FDA-approved drugs for treating *BRAF V600E*-mutated melanoma) obtained promising results in thyroid cancers with this mutation. Vemurafenib also proved capable of restoring radio-iodine uptake, to some degree at least^61^. As discussed earlier, however, few cases of metastatic thyroid disease are associated with *BRAF V600E* mutations, so other therapeutic targets are needed. Given the frequency of its mutations in metastatic cancers, and their prognostic role in thyroid cancer, the *TERT* promoter could be a future therapeutic target. Telomerase inhibitors (e.g. Imetelstat^62^), drugs inducing telomere dysfunction (e.g. 6-thio-2′-deoxyguanosine^63^), and adenoviral gene therapies that induce telomerase promoter-driven oncolytic activity^64^ are currently under investigation as potential anticancer agents. Based on the latest literature (as confirmed by the present study), telomerase could be a promising therapeutic target for RAI-refractory thyroid cancer, but no data are available as yet.

Our study confirms the prognostic impact of distant metastases, but not primary cancer size on patient outcomes. From the available literature, it is still hard to say whether large tumor size has an impact on patients’ prognosis. Our data suggest that larger DTCs differ in their molecular features from smaller ones, but are not more aggressive. We found a higher prevalence of *TERT* promoter mutations in patients with metastatic disease. This mutation confirmed its association with tumor aggressiveness, and was the only molecular event capable of significantly and independently influencing DTC outcome.

## Methods

### Patients

We selected 240 consecutive patients with a histological diagnosis of DTC who underwent total thyroidectomy from 2007 to 2016, and were followed up by the Endocrinology and Radiotherapy Units in Padua. We first ensured that adequate frozen material was available in the Tissue Bank after first surgery for all cases. Then patients were grouped as follows: group 1, DTC with the largest tumor focus ≤ 40 mm, without distant metastases; group 2, DTC with the largest tumor focus > 40 mm, without distant metastases; and group 3, metastatic DTC at diagnosis or detected during the follow-up, regardless of tumor size.

Clinical data were obtained from the electronic medical records. Surgical pathology specimens were analyzed by two expert pathologists (FG and GP). All pathological samples were reviewed and a histological diagnosis was established following the 4th edition of the WHO classification (WHO-2017)^65^. Pathological staging was done according to the 8th edition of the TNM staging system^8^. If there were multiple foci, the largest tumor dimension was considered.

All patients underwent total thyroidectomy and RAI treatment (median dose 200 mCi). When further treatments were indicated, these involved surgery, RAI treatments, external beam radiation, bisphosphonates (in cases of multiple bone lesions), and tyrosine kinase inhibitors (in cases of progressive metastatic RAI-refractory disease).

Following recent ATA guidelines^6^, we identified four possible outcomes, defining response as: excellent; biochemical incomplete; structural incomplete; or indeterminate. The median patient follow-up was 79.8 months (12.9–237 months).

This study was conducted according to the guidelines laid down in the Declaration of Helsinki. All patients participating in the study gave their written informed consent. The Ethical Committee for Clinical Experimentation at Padua Hospital approved the study protocol (Ref. 121).

### DNA extraction and mutation analysis

Genomic DNA was extracted from frozen tissues after surgery using the DNeasy Blood and Tissue kit (Qiagen, Italy), according to the manufacturer’s protocol. Mutation analyses were performed in all patients for *BRAF* (NM_004333.4). and for *N-RAS* (NM_002524.3; exons 2 and 3), *K-RAS* (NM_033360.2; exons 2 and 3) and *H-RAS* (NM_005343.2; exons 2 and 3). *TP53* (exons 5, 6,7 and 8) in 133/144 (92.4%) patients in group 1, 44/50 (88%) in group 2, and 44/46 (95.6%) in group 3. *PTEN* (exons 5, 7 and 8) was tested in all patients in groups 1 and 2, and in 45/46 (97.8%) in group 3. *PIK3CA* (exons 9 and 20) was tested in all patients in group 1, 43/50 (86.0%) in group 2, and 40/46 (86.9%) in group 3. The *TERT* proximal promoter (NM_198253.2) was tested in 133/144 patients (92.4%) in group 1, and in all patients in groups 2 and 3, by direct sequencing (ABI PRISM 3130, Applied Biosystems, Foster City, CA), as previously described elsewhere^66,67^.

### Statistical analysis

Categorical data were summarized using frequencies and percentages. Distributions of the continuous variables were assessed, and data were summarized accordingly. The comparison of continuous variables (age at diagnosis, tumor size, follow-up) between the three groups was done with the Kruskal–Wallis test. Group comparisons of the categorical variables were done with the χ^2^ test. A *p* < 0.05 was considered statically significant. DFS and CSS were calculated with the Kaplan–Meier method. A multivariate analysis was conducted with a logistic regression analysis. The Med Calc software version 19.1.7 (MedCalc Software Ltd, Ostend, Belgium; https://www.medcalc.org; 2020) was used for the statistical analysis.
